# Adsorption of phenylurea herbicides by tropical soils

**DOI:** 10.1007/s10661-020-8160-2

**Published:** 2020-03-04

**Authors:** Babatunde Kazeem Agbaogun, Klaus Fischer

**Affiliations:** Department of Analytical and Ecological Chemistry, University of Trier, Trier, Germany

**Keywords:** Tropical soil, Phenylurea herbicides, Adsorption, Soil properties, *K*_f_, Molecular descriptors

## Abstract

The distribution of pesticides in soils with consequences for their mobility, bioavailability and water contamination is mainly ruled by sorption processes. Such processes are seldom investigated in tropical soils. Thus, specific interactions between tropical soils and most pesticides are widely unknown. Furthermore, the question arises whether the same factors govern adsorption in tropical and temperate soils. Thus, the sorption behaviour of five phenylurea herbicides (PUHs) was studied in eighteen differently composed soils originating from southwestern Nigeria. Sorption data were obtained by equilibrating the soil samples with 0.01 M CaCl_2_ solutions spiked with increasing concentrations of the target PUHs. The equilibrium data fitted well to the Freundlich isotherm equation (*R*^2^ ≥ 0.96), delivering the corresponding parameters (*K*_f_ and *n*). Linear distribution coefficients (*K*_d_) were also calculated. The Pearson correlation was used to identify the specific soil and herbicide properties that have statistically significant correlations with sorption parameters. High correlations were established for various soil properties (pH, cation exchange capacity, organic carbon content, content of amorphous Fe and Mn oxides, clay/silt mass proportions) as well as molecular descriptors (octanol-water partition coefficient (log *K*_ow_) and molecular mass (Mw)) of the moderately hydrophobic herbicides. Monuron, chlorotoluron and isoproturon showed higher affinities for soil than previously reported. The gathered knowledge might assist in the assessment and in the precautionary avoidance of potential risks generated by these compounds in tropical soils.

**Electronic supplementary material:**

The online version of this article (10.1007/s10661-020-8160-2) contains supplementary material, which is available to authorized users.

## Introduction

Understanding the fate of pesticides in soil is fundamental for the accurate assessment of their environmental risk. It is also essential for the assurance of the safe use of both newly developed and legacy pesticides. The fate of pollutants in the environment is affected by many biogeochemical processes—a complex web of various physical, chemical and biological interactions (Ghafoor et al. 2011) where sorption on organic and mineral surfaces plays a key role. Sorption phenomena ultimately determine the availability of pollutants for biological and chemical degradation in soils (Karickhoff 1984; Murphy and Zachara 1995; Olvera-Velona et al. 2008). On the other hand, the degree to which a pesticide is adsorbed by soil itself is affected by many pedological and pesticide physicochemical properties (Weber et al. 2004; Olvera-Velona et al. 2008; Sanchez-Bayo and Hyne 2011; Hall et al. 2015). The interrelationships among these factors and their effects on sorption are still not fully understood, hence the continued research interest.

Among the most important and extensively used classes of pesticides worldwide are the phenylurea herbicides (PUHs) (El-Nahhal et al. 2013). PUHs have been commercialised for more than 50 years (Giacomazzi and Cochet 2004; Green and Young 2006; Baskeyfield et al. 2011). Most often, they are used for pre- or post-emergence control of weeds in diverse cropped areas and vineyards (Chhokar et al. 2008; Wang et al. 2015) and also in non-cropped areas such as roads, railway tracks and homes (Sørensen et al. 2003; Giacomazzi and Cochet 2004; Silkina et al. 2009). Diuron, in particular, is also used as algicide in antifouling paints. As a consequence of their wide use, PUHs have been detected in several environmental compartments as well as in food products around the world (Wang et al. 2015; Lu et al. 2018). Besides the PUHs themselves, some of their metabolites such as 3,4-dichloroaniline (3,4-DCA), *N*-(3,4-dichlorophenyl)urea (DCPU) and *N*-(3,4-dichlorophenyl)-*N*-methylurea (DCPMU) have also been detected in natural waters (Eriksson et al. 2007). Some evidence exists that PUHs exert adverse effects in aquatic organisms, small mammals and humans (Blondel et al. 2013; Lu et al. 2018). Specifically, isoproturon, diuron and linuron are suspected to evoke carcinogenic, mutagenic, teratogenic, endocrine-disrupting and cytogenetic effects in animals as well as humans (Orton et al. 2009; Lu et al. 2018). Some of their metabolites have also been reported to be much more harmful to non-target organisms than their parent compounds (Eriksson et al. 2007). Consequently, four PUHs—diuron, chlorotoluron, isoproturon and methabenzthiazuron—were included in the European Commission’s list of priority substances for European freshwater resources. Diuron, linuron, fluometuron and neburon were also listed on the US Environmental Protection Agency’s Second Drinking Water Contaminant Candidate (Sørensen et al. 2003; Federico et al. 2014).

Thus, concerns about the environmental behaviour of PUHs have increased over the years, raising many scientific issues. Several studies investigated their sorption behaviour in soils (Hance 1965; Grover 1975; Fouque-Brouard and Fournier 1996; Sanchez-Camazano et al. 2000; Liyange et al. 2006; Inoue et al. 2006; El-Khattabi et al. 2007; Fernandez-Bayo et al. 2008; Sanchez-Bayo and Hyne 2011; Elgouzi et al. 2012; El-Nahhal et al. 2013; Mendes et al. 2014; Hall et al. 2015). Also, Blondel et al. (2013) and Langeron et al. (2014) investigated the molecular properties that affect the adsorption coefficient of these herbicides. Nevertheless, most of these studies focused on temperate soils. Furthermore, almost all screening tools to evaluate the fate or mobility of herbicides in soils work with *K*_d_ and/or *K*_oc_ values derived from temperate soils (Rao et al. 1985; Mendes et al. 2014; Hall et al. 2015). This procedure is not justified as long as the following central question remains unanswered: are distribution coefficients from the temperate soils directly transferable to tropical soils despite the differences in soil types, climate conditions and cropping systems? Currently, no data exists on the phase distribution of PUHs in Nigerian and most other tropical soils to answer this question. Secondly, there are some research gaps regarding the importance of soil physicochemical properties for PUH adsorption, leaving the rules that control the process undiscovered. Therefore, the specific objectives of this study were (i) to determine the sorption behaviour of five commonly used PUHs (linuron, diuron, monuron, chlorotoluron and isoproturon) in a wide range of Nigerian soils, (ii) to identify the physicochemical controls of Nigerian soils on PUH sorption and (iii) to compare the sorption behaviour of these compounds in these soils with the available data, especially from temperate soils. Furthermore, considering the rather hydrophobic and non-ionic nature of PUHs and the body of work pointing out the dominance of soil organic matter (SOM) in their adsorption, we also evaluated the characteristics of PUH sorption in some organic matter–free (OMF) soils. The latter being an attempt to estimate the separate contributions of soil organics and minerals to sorption of PUHs in soils. We believe this knowledge will contribute to the potential use of organic matters, more importantly in solving problems associated with the pollution of ground and surface waters by fairly hydrophobic pesticides.

To achieve these objectives, the experimental studies were focused on the (1) measurement of sorption coefficients of the five PUHs in eighteen contrasting whole soils, (2) measurement of sorption coefficients of two PUHs (linuron and diuron) in few of the soil samples after SOM removal treatment and (3) measurement of sorption kinetics of three PUHs (linuron, diuron and monuron) in twelve whole soils. The results were subjected to statistical analysis against a wide range of soils and PUH properties to identify the trends and correlations. Soil as a three-dimensional body reflects, most especially, the impact of climate. Therefore, given the wide difference between temperate and tropical climates, it was assumed that PUH sorption in Nigerian (tropical) soils is different from that of temperate soils. It was also hypothesised that apart from SOM, soil mineral fractions contribute to PUH sorption. Lastly, based on the physicochemical properties of PUHs, the effect of SOM on sorption was expected to increase with increasing log *K*_ow_.

## Materials and methods

### Chemicals

**Fig. 1:**
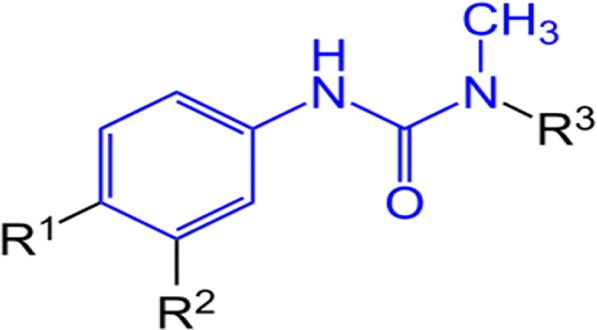
General structure of PUHs

Unlabelled analytical standards (99% purity) of diuron (3-(3,4-dichlorophenyl)-1,1-dimethyl-urea), linuron (3-(3,4-dichlorophenyl)-1-methoxy-l-methylurea), monuron (3-(4-chlorophenyl)-1,1-dimethyl-urea) and chlorotoluron (3-(3-chloro-4-methylphenyl)-1,1-dimethylurea) were obtained from Sigma-Aldrich (Germany), while isoproturon (3-(4-isopropylphenyl)-1,1-dimethylurea) was obtained from Dr. Ehrenstorfer (Augsburg, Germany). The chemical structures and properties of the selected PUHs are shown in Fig. 1 and Table 1. All other reagents were of analytical grade. PUH stock solutions (1 g L^−1^) were prepared in HPLC grade methanol, from where working solutions of 0.25–25 mg L^−1^ were prepared in 0.01 M CaCl_2_/Milli-Q water (membraPure, 0.055 μs/cm) solution.

**Table 1:** Common names, structures, properties and analytical parameters of the selected phenylureas

Common name	Abbr	CAS number	R^1^	R^2^	R^3^	log *K*_ow_	Mw^a^	*α*^b^	*S*^a^	log *S*	*λ*^c^	RT^c^
Diuron	DIU	330-54-1	Cl	Cl	CH_3_	2.60	233.1	26.2	36	1.56	249	8.70
Linuron	LIN	330-55-2	Cl	Cl	OCH_3_	3.00	249.0	27.2	64	1.81	248	10.25
Monuron	MON	150-68-5	Cl	H	CH_3_	1.81	198.0	23.9	230	2.36	246	5.13
Chlorotoluron	CLT	15545-48-9	CH_3_	Cl	CH_3_	2.51	212.0	26.1	74	1.87	241	7.22
Isoproturon	IPU	34123-59-6	(CH_3_)_2_C	H	CH_3_	2.50	206.3	28.1	70	1.85	243	6.54

log *K*_ow_ is the logarithmic value of octanol/water partition coefficient, *S* is the water solubility at 20 °C (mg L^−1^), log *S* is the logarithmic value of water solubility, *α* is the molecular polarisability (A^3^) and *λ* is the detection wavelength (nm)

*Abbr* abbreviation, *Mw* molecular weight (g mol^−1^), *RT* retention time (minute)

^a^Pesticide Properties Database (PPDB 2018)

^b^Gaussian calculation (Blondel et al. 2013)

^c^This study

### Soils

The soils stemmed from southwestern Nigeria, largely from the moist lowland and southern Guinea savannah ecological zones. The region is covered with dense forest and savannah vegetation (trees and shrubs), with and without canopy formation (Fagbemi and Shogunle 1995). This region has a temperature of between 28 and 32 °C (annual average), and a mean annual precipitation of 1000–1500 mm, with the rainy season lasting for 7–8 months. The geomorphology of this area is characterised by two major rock types: (i) arenaceous sedimentary rocks and deposits and (ii) crystalline basement complex rocks. Each rock type has its unique soils. But generally, the soils are ferruginous tropical soils with kaolinite as the dominant clay mineral. The dominant soil types in this region are Arenic Paleudalfs, Rhodic Paleudalfs, Oxic Tropudalfs, Typic Tropudults, Typic Tropaquepts, Oxic Paleudalfs, Oxic Paleustalfs, Aquic Tropopsamments and Typic Ustipsamments (pro parte) according to US soil taxonomy (Fagbemi and Shogunle 1995). These can be broadly classified as Luvisols, Lixisols, Gleysols, and Arenosols according to WRB classification (IUSS Working Group WRB 2014). As observed by Giresse (2008), almost all the tropical soils are fairly represented in the western part of the African continent, hence the choice of this study location.

Eighteen top soils (0–20 cm) with contrasting characteristics, and representing these major soil types, were collected at different agricultural and non-agricultural fields with the aid of a soil auger. The samples were air-dried, passed through a 2-mm sieve and stored at room temperature prior to use. Samples were extensively characterised using standard methods. Briefly, soil pH was measured in 1:2.5 (w/v) soil/0.01 M CaCl_2_ solution, with a pH meter. Soil organic carbon (SOC) was determined as the difference between soil total carbon (STC) and soil inorganic carbon (SIC). STC determination was based on complete oxidation of the carbon in previously crushed and oven-dried samples. Measurement was done using an Elemental Analyser EA3000 apparatus (HEKAtech GmbH, Wegberg, Germany). SIC for the samples with pH_CaCl2_ > 6.6 was determined by destruction of the soil carbonates with 50% phosphoric acids and by simultaneous measurement of the CO_2_ evolved by CHNS Elemental Analyser (HEKAtech GmbH, Wegberg, Germany). The effective cation exchange capacity (ECEC) was determined as the sum of extractable cations (Na + Ca + Mg + Na + K + Fe + Mn + Al) obtained after displacement with 1 M ammonium chloride solution (Shuman and Duncan 1990). Determination of the pedogenic metal oxides (Fe_d_, Al_d_ and Mn_d_) followed the dithionate-citrate extraction protocol of Mehra and Jackson (1960), while that of the active or amorphous proportions (Fe_o_, Al_o_ and Mn_o_) followed the acid ammonium oxalate extraction protocol of Tamm (1922), as modified by Schwertmann (1964). Here, metal oxides refer collectively to Fe, Al and Mn oxides, (oxy)hydroxides and hydrated oxides. Particle size distributions were determined using the laser diffraction technique, with Malvern Mastersizer 3000 (Malvern Instrument, UK). Briefly, samples (about 2 mg) were dispersed in 0.1 M Na-pyrophosphate for 12 h, followed by 15 min of ultrasonication. The suspensions were then transferred to the dispersion unit of the Mastersizer from where aliquots were taken under constant stirring for measurement. Each aliquot was measured five times (at a measuring time of 5 s per measurement, refractive index of 1.78, adsorption index of 0.1, assumed density of 2.6 g cm^−3^ and 8% obscuration), and each sample was measured in triplicate. An internal standard, nepheline thionate (Minex S4), was similarly measured at interval, for quality control. The scattering data were acquired and processed with Mastersizer 3000 software (Malvern Instrument, UK). Soil mineral identification was done by X-ray diffractometry (XRD) for the particle size fraction < 2 μm, using Siemens D500 diffractometer with Cu-K_α_ radiation. Bulk soil mineralogy was determined on air-dried powder preparations (random mounts), while clay mineralogy was determined on oriented specimens. Five XRD scans were run (one for each powder specimen and four for each oriented preparation), i.e. under dry air condition, after ethylene glycol (EG) solvation, under dimethyl sulfoxide (DMSO) solvation and after heating up to 550 °C. These four scans were necessary to differentiate between the four main clay groups: smectite, illite, chlorite and kaolinite. Data sets of powder and oriented diffraction files were compiled and executed with the Bruker AXS XRD software package DIFFRAC^plus^. Samples diffractograms were analysed by the DIFFRAC^plus^ Evaluation (EVA) programme (version 6.0 rev o), and Eq. (1) proposed by Biscaye (1965) was applied to obtain the semi-quantitative relationships between the different clay minerals.

1 $$ \mathrm{Area}\ 17\ \overset{{}^{\circ}}{\mathrm{A}}\ \left(\mathrm{EG}\right)+4\times \mathrm{area}\ 10\ \overset{{}^{\circ}}{\mathrm{A}}\ \left(\mathrm{EG}\right)+2\times \mathrm{area}\ 7\ \overset{{}^{\circ}}{\mathrm{A}}\ \left(\mathrm{air}\right)=100\% $$

where area 17 Å (EG) corresponds to percentage smectite, 4 × area 10$\overset{{}^{\circ}}{\mathrm{A}}$ (EG) corresponds to percentage illite and 2 × area 7$\overset{{}^{\circ}}{\mathrm{A}}$ (air) corresponds to percentage kaolinite + chlorite.

Statistical test results of variability, i.e. mean, coefficient of variation (CV), minimum (min) and maximum (max) of each soil property, were calculated with Excel^®^ (2013) spreadsheet. The physicochemical properties of the samples as well as the statistical test of variability are presented in Table 2.

**Table 2:** Physical and chemical properties of the soils

Soil	pH	C_inorg_ (%)	C_org_ (%)	%N	Ca	RCO	ECEC	Metal oxides (g kg^−1^)						Clay minerals			Particle size (%)			Textural class	USDA (orders)	Sampling coordinates
								Fe_d_	Al_d_	Mn_d_	Fe_o_	Al_o_	Mn_o_	ill	kao	sme	Clay	Silt	Sand			
Apm	5.4	nd	0.7	0.1	14	4.1	21.5	5.1	0.4	0.5	0.4	0.4	0.2	17	83	0	2.9	25.3	71.8	Sandy loam	Alfisol	N 7° 22.44′; E 3° 50.29′
Iwo	6.4	nd	1.3	0.1	41	2.2	49.8	7.5	0.6	0.8	0.4	0.6	0.3	59	41	0	2.9	38.3	58.8	Sandy loam	Alfisol	N 7° 22.56′; E 3° 50.34′
Egb	5.9	nd	1.0	0.8	29	3.9	39.3	11.5	0.5	1.2	0.8	0.5	0.3	17	83	0	3.9	36.9	59.2	Sandy loam	Alfisol	N 7° 22.08′; E 4° 04.52′
Mtk	5.9	nd	1.0	0.9	28	3.1	42.8	4.4	0.2	0.5	1.1	0.2	0.3	16	72	12	3.1	33.4	63.6	Sandy loam	Alfisol	N 7° 22.73′; E 3° 50.47′
Skn	7.5	0.1	1.4	0.1	67	3.0	78.5	11.7	0.6	1.5	0.7	0.6	0.9	18	82	0	4.1	42.5	53.4	Sandy loam	Inceptisol	N 7° 38.18′; E 4° 26.70′
Klb	5.1	nd	0.6	0.0	23	3.4	30.5	2.4	0.1	0.2	0.6	0.1	0.2	7	85	8	2.0	22.3	77.7	Loamy sand	Entisol	N 7° 53.01′; E 3° 52.89′
Mny	6.6	nd	1.1	0.1	32	4.0	47.8	9.7	0.6	1.3	0.8	0.6	0.6	8	92	0	4.3	35.6	66.1	Sandy loam	Alfisol	N 7° 34.33′; E 3° 55.15′
Gbn	7.2	0.0	1.4	0.1	50	2.7	68.9	8.4	0.2	1.1	0.5	0.3	0.4	49	51	0	3.9	45.8	50.3	Sandy loam	Inceptisol	N 7° 29.78′; E 4° 21.76′
Akn	6.6	nd	1.4	0.1	46	3.0	59.5	8.9	0.5	0.9	0.6	0.5	0.3	14	86	0	4.2	46.8	49.0	Sandy loam	Alfisol	N 7° 21.35′; E 3° 56.06′
Odd	5.9	nd	0.5	0.1	20	4.5	27.6	2.0	0.3	0.0	0.3	0.4	0.2	18	82	0	2.4	24.1	73.5	Loamy sand	Alfisol	N 7° 14.78′; E 3° 32.45′
Iwr	6.3	nd	0.8	0.1	37	3.5	45.7	3.6	0.3	0.2	0.3	0.4	0.2	36	64	0	2.8	32.6	64.6	Sandy loam	Inceptisol	N 7° 39.31′; E 3° 47.83′
Ibd	5.7	nd	0.5	0.5	13	6.6	22.3	9.4	0.6	1.0	0.5	0.6	0.2	23	77	0	3.5	31.6	68.4	Sandy loam	Alfisol	N 7° 21.22′; E 3° 49.54′
Ond	5.8	nd	0.9	0.1	17	4.5	27.8	7.2	0.4	1.0	0.4	0.4	0.3	8	92	0	3.8	31.6	64.6	Sandy loam	Alfisol	N 7° 37.92′; E 3° 55.28′
Idt	5.7	nd	0.8	0.1	64	3.1	74.0	2.0	0.2	0.0	0.2	0.2	0.1	49	51	0	2.5	25.6	73.1	Loamy sand	Entisol	N 7° 56.01′; E 3° 40.56′
Uib	7.1	0.2	4.0	0.4	90	1.5	111.7	13.7	0.6	3.1	1.9	0.5	2.3	22	78	0	6.0	59.0	35.0	Silt loam	Inceptisol	N 7° 26.87′; E 3° 53.88′
Asj	6.5	nd	1.2	0.1	64	3.7	67.0	14.6	0.3	2.4	0.9	0.3	0.3	21	76	3	4.3	48.5	47.2	Sandy loam	Inceptisol	N 7° 21.29′; E 4° 08.08′
Bkt	5.8	nd	1.9	0.5	39	2.4	89.6	12.6	0.4	1.8	0.4	0.4	0.2	7	93	0	4.7	43.2	52.1	Sandy loam	Alfisol	N 7° 23.72′; E 3° 42.25′
Fas	5.6	nd	0.8	0.1	20	3.1	26.8	14.4	0.3	2.4	1.1	1.4	0.4	17	83	0	2.5	26.5	73.5	Loamy sand	Alfisol	N 7° 54.01′; E 3° 46.81′
Min	5.1	0.0	0.5	0.0	13	1.5	21.5	2.0	0.1	0.0	0.2	0.1	0.1	7	41	0	2.0	22.3	35.0			
Max	7.5	0.2	4.0	0.9	90	6.6	111.7	14.6	0.6	3.1	1.9	1.4	2.3	59	93	12	6.0	59.0	77.7			
CV	10.3	nd	65.2	111.1	54.3	31.4	48.1	50.3	40.1	77.4	61.9	57.9	116.5	66.9	19.2	258.1	27.5	26.9	18.2			

Ca and ECEC are expressed in mmol_c_ kg^−1^;

*sme* smectite, *ill* illite, *kao* kaolinite, *nd* not determined, *CV* coefficient of variation, *RCO* clay-to-organic carbon ratio

### Soil organic matter removal

Chemical destruction of soil organic matter was done to expose mineral surfaces for subsequent sorption experiments. This was carried out according to a modified method of Lavkulich and Wiens (1970), in sequential (5-cycle) oxidation with hot 3 M NaOCl (14% active Cl). After the complete treatment, the pH of the samples was controlled and readjusted repeatedly until the final pH of the residues was the same as those of the original soils. Finally, the residues were washed several times with deionised water and air-dried. The samples were subsequently labelled as omf (organic matter free). This method was used particularly as it favours significantly minimal modifications of the soil mineral constituents (Lavkulich and Wiens 1970; Mikutta et al. 2005).

### Sorption experiments

All sorption experiments were carried out using the batch equilibrium method, according to the OECD 106 guideline (OECD 2000). For the sorption isotherm experiments, soil (2 g dry weight (dw) per sample) was weighed into 50-mL polypropylene tubes, to equilibrate with 20 mL of 0.01 M CaCl_2_/Milli-Q water solutions of each pesticide, at concentrations ranging from 0.25 to 25 mg L^−1^. The suspensions were continuously shaken (with an end-over-end shaker, at 8 rpm) at 20 °C ± 1 °C for 24 h in the dark and then centrifuged at 4000 rpm for 15 min. For sorption kinetic experiments, soil (1 g dw per sample) was weighed into 50-mL polypropylene tubes, to equilibrate with 10 mL of 20 mg L^−1^ (for linuron and diuron) and 10 mg L^−1^ (for monuron), in 0.01 M CaCl_2_/Milli-Q water solutions. The suspensions were continuously shaken and subsequently treated as described above, with a set of tubes withdrawn at time intervals (20 min, 40 min, 90 min, 180 min and 360 min) to obtain sorption data at different periods of time. In both experiments, the supernatants were filtered with 0.2-μm Phenex RC membrane filters (Phenomenex, Torrance, USA), and the filtrate was collected into 2-mL amber LC vials and measured immediately with HPLC or kept in refrigerator at 4 °C (for a maximum of 2 days) prior to analysis. All experiments were conducted in duplicates and at soil natural pH. Since the results of several pre-studies showed very low data variability, this number of parallels is justified. Blank samples and control tests (in the same tubes and conditions as the sorption tests) were also run in parallel as quality control measures. From preliminary investigations, no significant adsorption of the test compounds occurred on the experimental tubes, neither were residues of the test compounds found in the experimental soils. Apart from their reported half-lives (in soils) extending far beyond the test period, compounds were also established to be relatively stable during the sorption experiment from repeated preliminary tests.

### Pesticide analysis

Analyses were carried out with a Shimadzu 10ADvp HPLC system (Shimadzu, Duisburg, Germany), equipped with a photodiode array detector (SPD-M20A, Prominence). The column was a C_18_ reversed-phase Hypersil (length, 250 mm; inner diameter, 4 mm; particle size, 5 μm; Thermo Fisher Scientific, Dreieich, Germany), and the sample injection volume was 20 μL. An isocratic eluent, composed of methanol/2.5% acetic acid Milli-Q water solution (60/40 v/v), was applied at a flow rate of 0.5 mL min^−1^. Other parameters are presented in Table 1. Data were collected and processed with LabSolutions software (Shimadzu, Duisburg, Germany). Quantification was based on seven-point calibration functions covering a concentration range of 0.1–25 mg L^−1^ for each analyte. The achieved coefficients of determination (*r*^2^) were greater than 0.99. All samples were analysed in triplicate, and the within-runs CVs were below 5%.

### Evaluation of sorption data

It was assumed that the differences between the added concentrations (*C*_0_, mg L^−1^) and the equilibrium concentrations in the aqueous phase (*C*_e_, mg L^−1^) (directly determined from the HPLC measurements) were solely due to sorption. Therefore, the amount adsorbed by the solid phase (*Q*_e_, mg kg^−1^) was calculated based on mass balance as follows:

2 $$ {Q}_{\mathrm{e}}=\left(\frac{C_0-{C}_{\mathrm{e}}}{m_{\mathrm{s}}}\right)\times V $$

where *V* (L) is the volume of the solution, and *m*_s_ is the mass of the soil (kg).

The percentage sorbed of PUH (*S* (%)) was calculated as

3 $$ S\ \left(\%\right)=\left(\frac{C_0-{C}_{\mathrm{e}}}{C_0}\right)\times 100 $$

The linear distribution coefficient of the sorption (*K*_d_, L kg^−1^) was calculated as

4 $$ {K}_{\mathrm{d}}={Q}_{\mathrm{e}}/{C}_{\mathrm{e}} $$

This was normalised on the basis of SOC to obtain *K*_oc_ (i.e. *K*_oc_ = *K*_d_ / *f*_oc_), where *f*_oc_ (soil organic carbon fraction) is given as SOC/100.

Further, the *K*_d_ of organic compounds in soils was expressed as the sum of two contributions: *K*_d.mineral_ that accounts for sorption by soil minerals (i.e. all soil constituents other than SOM), which could be significant in soils with low SOM, and *K*_d.organic_ that corresponds to (ad)sorption by the SOM (Millinovic et al. 2015; Delle Site 2001). The relationship is presented in Eq. (5).

5 $$ {K}_{\mathrm{d}.\mathrm{soil}}={K}_{\mathrm{d}.\mathrm{organic}}+{K}_{\mathrm{d}.\mathrm{mineral}}={K}_{\mathrm{oc}}\times {f}_{\mathrm{oc}}+{K}_{\mathrm{d}.\mathrm{mineral}} $$

Arising from this expression, the average value of *K*_oc_ for a given PUH was derived from the slope of *K*_d.soil_ vs *f*_oc_, while the intercept on the *y*-axis gave an estimate of the average *K*_d.mineral_.

Sorption isotherms were constructed by plotting *Q*_e_ vs *C*_e_ for each PUH-soil combination. Also, isotherm data were fitted to the Freundlich model which is the most frequently used model for hydrophobic compounds (Hinz 2001). The Freundlich model is quantitatively described by Eqs. (6) and (7).

6 $$ {Q}_{\mathrm{e}}={K}_{\mathrm{f}}.{\left({C}_{\mathrm{e}}\right)}^n $$

which transforms to the logarithmic form

7 $$ \log\ {Q}_{\mathrm{e}}=n\ \log\ {C}_e+\log\ {K}_{\mathrm{f}} $$

where *n* is a dimensionless empirical parameter that provides an indication of the isotherm linearity (i.e. *n* = 1 for a linear isotherm), and *K*_f_ is the specific Freundlich sorption coefficient expressed as (mg kg^−1^) (mg L^−1^)^−*n*^. *K*_f_ and *n* were calculated by linear least squares fitting of the sorption isotherms. *K*_f_ was also normalised on the basis of the SOC to obtain *K*_foc_ (i.e. *K*_foc_ = *K*_f_ / *f*_oc_).

To mathematically describe the intrinsic kinetic adsorption mechanisms, the kinetic data were fitted to two widely applied models, i.e. pseudo-first-order (PFO) and pseudo-second-order (PSO) (Eqs. (8) and (9), respectively).

8 $$ \log\ \left({Q}_{\mathrm{e}}-{Q}_t\right)=-\frac{k_1}{2.303}t+\log\ {Q}_{\mathrm{e}} $$

9 $$ \frac{t}{Q_t}=\left(\frac{1}{Q_{\mathrm{e}}}\right)t+\frac{1}{k_2{Q_e}^2} $$

where *Q*_*t*_ is the amount of pesticide adsorbed (mg kg^−1^) at any time (*t*, min); *k*_1_ (min^−1^) and *k*_2_ (kg mg^−1^ min^−1^) are the rate constants of PFO and PSO, respectively; and *Q*_e_ is as previously described. The initial adsorption rate (*h*, mg kg^−1^ min^−1^) is given by *k*_2_*Q*_e_^2^.

All calculations, plots and development of regression equations were done with Microsoft Excel^®^ (2013), while correlation analysis and test of significance were done with R-project open source software. Specifically, linear and multiple correlations were performed between the selected pesticide properties and the sorption coefficients (*K*_f_ and *K*_d_), between the sorption coefficients and the soil properties and among soil properties themselves, using the Pearson product moment correlation (PPMC). The Pearson correlation coefficient (*r*) was calculated as Eq. (10). Also, the goodness of fit of the regression equations was determined with the coefficient of determination (*R*^2^), given as Eq. (11).

10 $$ r=\frac{n\left(\sum xy\right)-\left(\sum x\right)\left(\sum y\right)}{\sqrt{\left[n\sum {x}^2-{\left(\sum x\right)}^2\right]}\left[n\sum {y}^2-{\left(\sum y\right)}^2\ \right]} $$

11 $$ {R}^2=1-\frac{\sum \limits_i{\left({x}_i-{y}_i\right)}^2}{\sum_i{\left({x}_i-\frac{1}{n}{\sum}_{i=0}^nx\right)}^2} $$

## Results and discussion

### Soil characteristics

Since one of the main goals of this study was to determine the most important soil-specific properties that influence sorption, we selected soils that represented a wide range of geochemical properties. The main physicochemical characteristics of the soils as well as their descriptive statistics are presented in Table 2. With the exception of Skn, Gbn, Uib, Iwo, Mny, Akn, Iwr and Asj which were slightly neutral (6.3–7.5), all other soils were acidic—with a pH range of 5.4 to 5.9. Organic carbon ranged from 0.5% (Ibd and Odd) to 4% (Uib). The ECEC ranged from 21.5 mmol_c_ kg^−1^ (Apm) to 111.7 mmol_c_ kg^−1^ (Uib). Uib also had the highest cumulative amount of pedogenic and free metal oxides (22.10 g kg^−1^), while Odd has the least amount (2.72 g kg^−1^). Particle size analysis showed that all the soils had low clay contents in the range 2.4–6.0%, while silt proportion ranged from 24.1 to 59%. Uib had the highest percentage of both clay and silt (6% and 59%, respectively), while Odd had the least of both (2.4% and 24%, respectively). XRD analysis revealed the presence of quartz, microcline and albite in all samples, while few samples contain sodalite, calcite and dolomite in addition (figures not shown). Semi-quantitative analysis of the clay indicated the presence of 2:1 expansive clay smectite (a compound name for montmorillonite, beidellite, nontronite and saponite) in only Mtk, Klb and Asj (at 12.3%, 7.9% and 2.6%, respectively), in addition to illite (a 2:1 non-expansive clay mica) and kaolinites (a 1:1 non-expansive clay) which were present in all soils. Generally, kaolinite and illite ranged from 7.4 to 59.4% and from 40.7 to 92.6%, respectively, in all soils. Using the United States Department of Agriculture (USDA) soil texture classification, thirteen (13) of the soils were sandy loam, four (Odd, Idt, Asj and Fas) were loamy sand, while only Uib was silty loam.

### Sorption kinetics

**Fig. 2:**
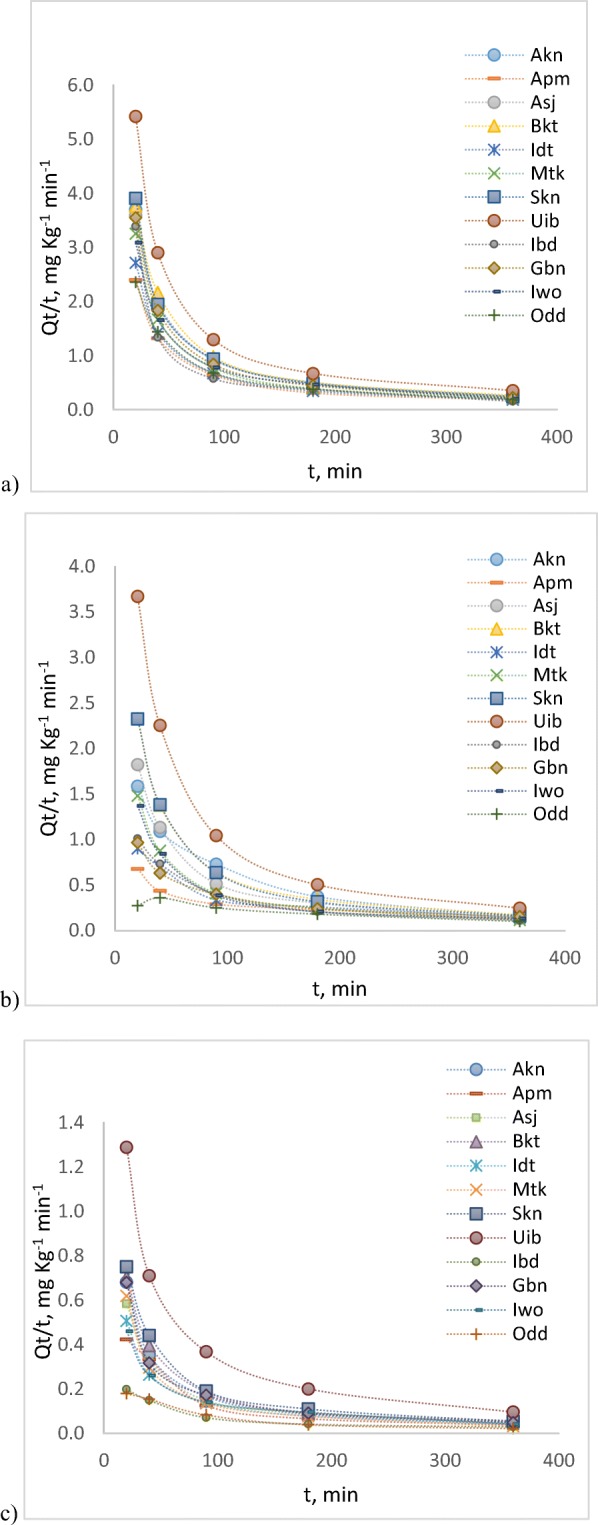
Sorption kinetic profiles of **a** linuron, **b** diuron and **c** monuron

Figure 2 a, b and c respectively displays linuron (LIN), diuron (DIU) and monuron (MON) sorption profiles with time, for twelve selected soils. Generally from the decline curves, a three-step kinetic process was observed: a rapid adsorption at the initial stage from *t* = 0 to *t* = 30 min, followed by a slow rate-limiting second stage and then the final long constant stage (reaching an apparent equilibrium between 120 and 180 min for the three compounds, in most of the soils). This observation is attributed to the heterogenous nature of soil (Morrica et al. 2000; Morrilo et al. 2000). Multi-stage sorption process of organic contaminants in soils/sediments has been reported (Pan et al. 2012). This initial fast adsorption suggests that PUHs may not undergo rapid seepage into the ground water upon discharge on the soil. The later slow adsorption would constitute the rate-determining step for the other (bio)geochemical interactions of the compounds with soils. Figure 2 further depicts that the adsorption rates decreased with time until equilibrium was attained at 180 min. However, we observed slight reversibility of LIN in few of the soils (especially Ibd) after 180 min. This observation may be connected to their SOM content. Ibd, for instance, has the least SOM. Thus, most of the sorption occurred on the soil minerals which are easier to desorb. The weak adsorption of non-ionic solutes on soil minerals may be attributed to the stronger competitive adsorption of water for the polar mineral surfaces (Chiou 2002). This often leads to the formation of outer-sphere surface complexes with generally less stable bonds (Sposito 2008).

PFO and PSO equations were used to fit the kinetic data in order to derive the kinetic parameters and to identify the kinetic mechanism(s) involved. However, only PSO showed very good fits, with *R*^2^ ranging from 0.96 to 1.0. Thus, only PSO parameters are selectively presented (supplementary material, SM1). It is thus observed that the theoretical *Q*_e_ (*Q*_e model_) fairly agreed with the experimental *Q*_e_, and both followed the trend of SOC, and log *K*_ow_ and Mw (of the compounds). The initial adsorption rate (*h*) also fairly correlated with log *K*_ow_ and the Mw of the compounds, but its trend with soil properties, most importantly SOC, was not readily discernible. No relationship was found between *k*_2_ and either the soil properties or the molecular descriptors (log *K*_ow_ and Mw).

### Sorption isotherms

The shapes of isotherm curves provide an overview of the relationship between sorbate and adsorbent at equilibrium (Giles et al. 1960). The isotherm plots of the five PUHs in all the soils are shown in SM2. Further, to confirm the linearity or otherwise of *Q*_e_ vs *C*_e_ and to deduce the sorption coefficients of each PUH-soil system, the isotherm data were fitted to the Freundlich equation. Table 3 shows the values of the sorption parameters (*n* and *K*_f_) obtained from the Freundlich fittings, as well as the *K*_d average_ (which is the mean of the *K*_d_ values for the six concentration levels). For all cases, satisfactory fittings were obtained with coefficient of determination (*R*^2^) ranging from 0.94 to 1.00 (figures not shown). The value of *n* represents a joint measure of both the relative magnitude and diversity of energies associated with a particular sorption process (Delle Site 2001). In this study, we observed *n* < 1 in all cases (Table 3). While it ranged from 0.3 to 0.4 for chlorotoluron (CLT), it was close to unity for the remaining four compounds. Isoproturon (IPU), in particular, had a very high *n* value (0.9) in Ond. Generally, *n* < 1 represents a convex, downward-curved, Langmuir-type (L-type) isotherm (Giles et al. 1960), and it is the most common type of sorption interactions between herbicides and soils (Petter et al. 2017). It indicates that the marginal sorption energy decreases with increasing surface concentration and often arises where the competition of solvent for sites is minimal or the adsorbate is a planar molecule (Delle Site 2001). *n* values close to 1 indicate a seemingly constant partitioning of the compounds in SOM. On the other hand, *n* values close to 0 indicate a predominance of adsorption onto soil minerals.

**Table 3:** Sorption parameters of the Freundlich equations for DIU, LIN, MON, CLT and IPU

PUH	Sorption parameters	Akn	Apm	Asj	Bkt	Ibd	Idt	Mtk	Odd	Skn	UIb	Gbn	Iwo	Klb	Mny	Iwr	Ond	Egb	Fas	Mean
DIU	*n*	0.7	0.7	0.7	0.7	0.7	0.6	0.7	0.7	0.6	0.7	0.7	0.8	0.6	0.7	0.7	0.7	0.7	0.7	0.7
	*K*_f_	11.9	7.4	8.0	10.0	5.4	9.3	10.3	5.3	14.1	18.9	9.7	6.9	8.0	7.5	8.4	7.8	8.1	5.7	9.0
	*R*^2^	1.00	0.99	0.99	0.98	0.98	0.97	0.98	0.96	0.99	0.99	1.00	1.00	1.00	1.00	1.00	0.99	1.00	1.00	0.99
	*K*_d average_	8.8	5.2	5.8	7.2	3.6	6.2	7.2	3.5	10.7	15.5	9.0	6.0	5.1	5.1	5.7	5.1	5.4	3.6	6.6
	*K*_oc_	625.2	744.2	496.0	375.5	674.6	770.7	729.2	659.0	786.0	387.1	631.3	462.7	870.0	477.7	712.6	595.7	528.2	434.5	608.7
	*K*_foc_	844.7	1054.9	685.4	521.0	1020.3	1142.1	1040.6	1006.1	1037.2	471.8	683.3	527.1	1352.2	697.9	1053.9	907.0	795.8	692.1	863
	*n*	0.7	0.7	0.8	0.7	0.8	0.7	0.7	0.8	0.7	0.7	0.8	0.8	0.6	0.7	0.7	0.7	0.7	0.7	0.7
	*K*_f_	14.3	9.2	10.8	12.7	5.9	10.9	12.0	6.0	17.1	28.1	12.3	8.1	9.2	9.9	9.8	9.9	8.8	6.8	11.2
LIN	*R*^2^	1.00	1.00	1.00	1.00	0.99	1.00	1.00	0.99	1.00	1.00	0.98	0.99	1.00	0.99	1.00	1.00	1.00	0.99	0.99
	*K*_d average_	11.1	6.6	8.2	9.7	4.7	8.0	8.9	4.8	13.9	25.5	9.8	6.4	6.4	7.2	7.1	7.1	6.4	4.6	8.7
	*K*_oc_	785.1	937.9	703.1	502.7	884.5	982.1	893.4	903.6	1020.0	635.2	688.9	489.5	1078.3	669.3	891.5	820.5	630.4	561.0	782.1
	*K*_foc_	1016.4	1312.8	922.4	663.7	1113.3	1342.2	1208.8	1138.2	1257.1	700.4	865.2	620.4	1564.8	922.6	1221.8	1145.3	861.0	833.5	1039.4
	*n*	0.7	0.7	0.9	0.9	0.7	0.6	0.7	0.7	0.7	0.8	0.8	0.7	0.7	0.6	0.8	0.8	0.8	0.8	0.7
	*K*_f_	4.9	3.6	2.5	3.2	2.4	4.8	4.4	2.4	6.2	9.4	3.6	2.7	3.6	3.9	2.8	2.6	2.7	1.7	3.7
MON	*R*^2^	1.00	0.99	0.99	0.99	1.00	0.98	0.98	1.00	1.00	1.00	0.99	0.99	0.98	1.00	0.99	0.98	0.98	0.93	0.99
	*K*_d average_	3.2	2.4	2.0	2.7	1.5	2.8	2.8	1.5	4.1	7.0	2.5	1.9	2.2	2.3	2.0	1.8	1.8	1.2	2.6
	*K*_oc_	227.9	348.3	173.9	139.8	290.6	346.5	284.4	290.6	304.6	173.6	177.3	145.4	367.8	212.4	255.5	206.6	180.3	149.3	237.5
	*K*_foc_	349.6	513.1	210.1	165.0	454.1	588.2	443.6	454.1	455.1	234.0	255.0	206.0	603.3	362.6	344.4	301.9	266.3	212.8	356.6
	*n*	0.3	0.3	0.4	0.3	0.3	0.3	0.3	0.3	0.3	0.3	0.3	0.4	0.3	0.3	0.3	0.3	0.3	0.3	0.3
	*K*_f_	7.5	4.8	5.4	7.3	2.8	5.1	5.9	3.3	9.5	15.8	5.4	3.6	4.1	4.2	5.0	4.2	4.1	4.2	5.7
CLT	*R*^2^	1.00	1.00	0.99	1.00	0.99	0.99	0.99	1.00	1.00	1.00	0.9	0.98	0.97	0.99	1.00	1.00	0.99	0.99	0.99
	*K*_d average_	5.4	3.5	4.1	5.7	2.0	3.6	4.1	2.3	7.2	13.1	4.3	2.8	2.7	3.0	3.5	2.9	2.6	2.6	4.2
	*K*_oc_	382.1	505.4	352.1	294.1	378.5	449.4	413.8	431.5	527.3	327.5	300.2	214.5	455.1	278.3	431.4	333.1	252.6	316.5	369.1
	*K*_foc_	532.2	689.8	461.2	381.0	533.5	634.9	597.5	628.2	700.3	394.5	383.0	279.8	694.3	396.3	628.5	492.0	404.4	507.1	518.8
	*n*	0.8	0.7	0.8	0.9	0.8	0.8	0.8	0.8	0.7	0.8	0.8	0.9	0.7	0.8	0.7	0.9	0.8	0.9	0.8
	*K*_f_	3.0	2.3	3.1	2.9	1.5	2.3	2.5	1.5	3.7	5.9	2.4	1.6	2.6	1.2	2.1	1.4	1.7	1.1	2.4
IPU	*R*^2^	1.00	0.99	1.00	0.98	0.99	0.99	1.00	0.97	1.00	0.99	1.00	1.00	0.96	0.97	0.99	0.98	0.99	1.00	0.99
	*K*_d average_	2.2	1.5	1.9	2.3	1.1	1.7	1.7	1.0	2.6	4.6	1.8	1.4	1.8	0.9	1.4	1.3	1.2	0.9	1.7
	*K*_oc_	158.2	215.6	165.8	120.1	215.3	206.3	176.2	186.6	189.0	113.7	126.8	103.5	301.7	84.6	177.0	153.7	116.8	106.8	162.1
	*K*_foc_	210.2	321.2	268.8	150.8	279.4	283.8	248.4	274.7	274.7	147.6	168.7	122.3	444.1	110.7	267.9	167.8	165.1	130.1	224.2

*K*_oc_ and *K*_foc_ (both in L kg^−1^) indicate organic carbon–normalised *K*_d_ and *K*_f_, respectively. The abbreviations (i.e. Akn, Apm, Asj, Bkt,…, Fas) refer to soil samples and were formed from the names of sampling locations

Based on the molecular structure (substituted aromatic ring), sorption of PUHs may result basically from hydrophobic interactions in solutions and from van der Waals/specific interactions in the sorbed phase (Spurlock and Biggar 1994). While hydrophobic interaction accounts for the adsorption by SOM which could be by π-stacking between the phenyl moiety and the soil surface, the following specific interactions with soil minerals and/or hydrophilic moieties are possible: (1) interaction between the double-bonded oxygen of the carbonyl and a hydrogen atom of the clay lattice, (2) hydrogen bonding between the NH group of the aryl ammonium ions and the silicate layer and (3) hydrogen bonding through the water bridge formed by the primary hydration shell of clay cation (i.e. between the organic compound and the clay complex). These specific interactions are directional in nature (i.e. site directed). Therefore, the value of the Freundlich number (*n*) also reflects the complex interplay between sorbate properties (polarity, size, shape) and sorbent properties (physical [e.g. steric] or chemical [e.g. electronic]). The further *n* deviates from unity—as it was found in CLT, the greater the degree of site-specific interaction (Rae et al. 1998).

**Fig. 3:**
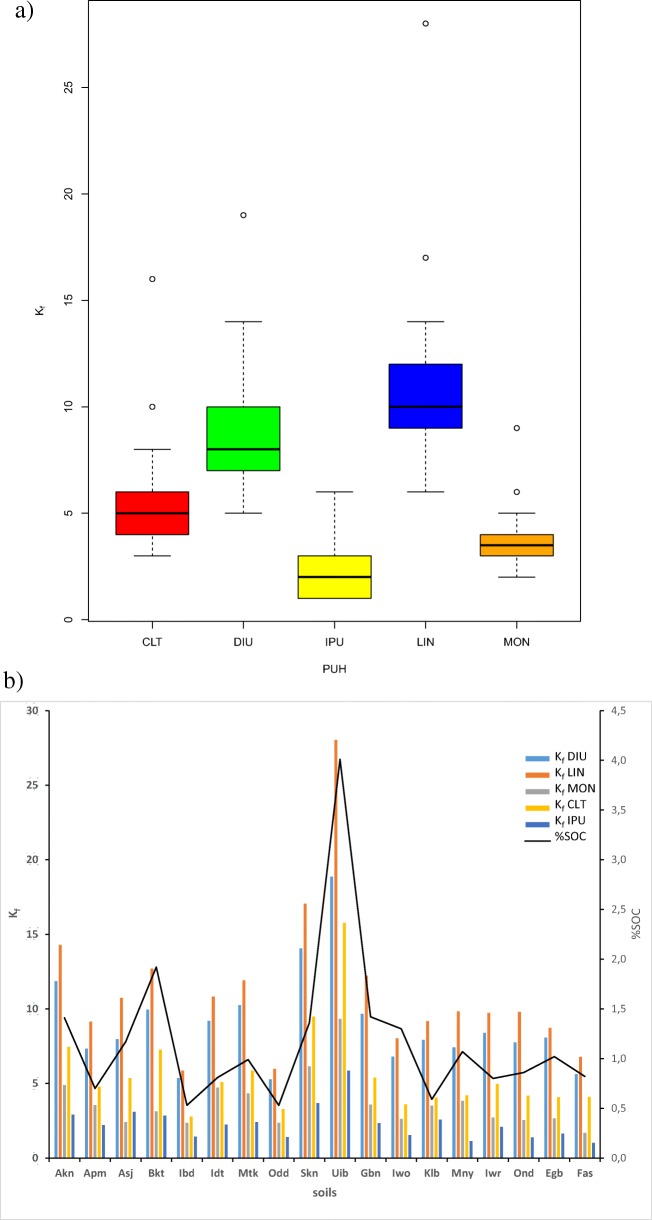
**a** Boxplots showing the mean *K*_f_ of the five compounds (the median is marked by the horizontal line inside the inner rectangle; the whiskers above and below the inner rectangle respectively represent the locations of the maximum and minimum, while the circles represent suspected outliers. **b** Charts of *K*_f_ and its trend with SOC

The sorption coefficients (*K*_d average_, *K*_oc_ and *K*_foc_) are also presented in Table 3. The *K*_d average_ values reported here are the means of six concentration levels. In all soils, LIN recorded the highest values for all the coefficients, followed by DIU, CLT, MON and IPU, in that order. The highest values of these parameters are associated with Uib which has the highest values of virtually all the soil parameters, while the least values alternate between Odd, Ibd and Fas (which are soils that have the lowest values of most of the parameters). The mean *K*_f_ of the five compounds and their trend with SOC are presented in Fig. 3 (a and b, respectively).

Basically, it could be observed that LIN has the highest *K*_f_ in all the soils, followed by DIU, CLT, MON and IPU, in that order. Also, it was observed that the highest *K*_f_ values correspond to soil (Uib) with the highest SOC, while the least values correspond to the soils (Odd and Ibd) with the lowest SOC (Fig. 3b).

Arising from the plots of *K*_d_ vs *f*_oc_ (SM3), we obtained the following regression equations: 564.74*x* + 1.98 for LIN, 328.68*x* + 2.71 for DIU, 305.81*x* + 0.56 for IPU, 144.01*x* + 0.85 for MON and 96.64*x* + 0.59 for CLT. Although the regression coefficients (*R*^2^) were not so close to unity (0.78–0.87), we could still fairly deduce *K*_oc_ values from the plots (Eq. (5)). Consequently, we found that LIN has the highest *K*_oc_ (565), followed by DIU (329), IPU (306), MON (144) and CLT (97). Expectedly, these values are respectively lower than our reported averages for these compounds (Table 3). However, except for CLT, the values gave fair estimates of the adjusted *K*_oc_; one may obtain after subtracting the *K*_d.mineral_ from the *K*_d.soil_. From the plots also, the order of the sorption to soil mineral was DIU > LIN > MON > CLT > IPU, with estimated *K*_d.mineral_ values of 2.71 (DIU), 1.98 (LIN), 0.85 (MON), 0.59 (CLT) and 0.56 (IPU). These values indicated that *K*_d.mineral_ contributed between 15 and 40% to the *K*_d average_ reported for these compounds (Table 3). In other words, it indicates that soil mineral fractions, vis-à-vis clay minerals and the amorphous metal oxides, also contributed fairly significantly (about 15–40%) to the sorption of the five test compounds in the soils. Sanchez-Camazano et al. (2000) have also reported significant adsorption of urea herbicides by clays. Nevertheless, Morrill et al. (1982) have reported that humic substances occur in intimate contact with other soil constituents, and that mineral-SOM interactions determine the adsorptive capacity of each soil. In fact, soil organic matters are said to exist as coatings on clay minerals, forming soil organo-clay complexes. The relationships between sorption coefficients, SOC and other soil physical and chemical properties were further explored in a later section.

### Sorption on organic matter–free soils

The positive relationship between pesticide sorption and SOM has been widely reported. Thus, SOM has been assumed to be the primary constituent responsible for inactivating pesticides (Weber et al. 2004). We also investigated the contribution of SOM to the sorption coefficients reported in our study. Table 4 shows the SOC of the selected 4 samples before and after organic matter removal treatment. It was observed that except in Uib where the SOC was reduced by only 75%, the treatment achieved between 93 and 96% reductions in the SOC of the other three soils.

**Table 4:** SOC, *K*_f_ and *K*_d_ for whole soils and omf

Samples	SOC ws	SOC omf	% SOC removed	*K*_f_ ws	*K*_f_ omf	% *K*_f_ reduction	*K*_d_ ws	*K*_d_ omf	% *K*_d_ reduction
Apm. DIU	0.7	0.05	93	7.4	0.7	91	5.2	0.6	89
Gbn. DIU	1.4	0.09	93	9.7	0.8	92	9.0	0.6	93
Ibd. DIU	0.6	0.03	94	5.4	0.5	90	3.6	0.5	86
Uib. LIN	4.3	1.06	75	29.2	6.7	77	28.4	5.4	81

*ws* whole soil, *omf* organic matter–free soil

It was also observed that the sorption coefficients for the treated soils were lowered by the corresponding percentage of the reduction in their SOC. This confirmed the dominating effect of SOM in the sorption of PUHs.

### Correlations between variables

#### Intercorrelations between soil basic parameters

The intercorrelations between the basic properties of the soils used in this study were described with Pearson correlation coefficients, and the results are presented in Table 5. In summary, pH was found to have positive and significant correlations with SOC, exchangeable Ca, ECEC, Al_d_, Mn_o_, %clay and %silt and a very significant negative correlation with %sand. SOC showed highly significant and strong positive correlations with exchangeable Ca, ECEC, Fe_d_, Fe_o_, Mn_d_, Mn_o_, %clay and %silt. Generally, soil cation exchange capacity (CEC) is largely determined by the charge of the soil particles and SOM (Morrill et al. 1982). Especially in the highly weathered tropical soils, where clay fraction is predominantly composed of oxides and hydroxides of Fe, Al and Mn (as in the present study) and kaolinite (1:1 silicate clays of low reactivity), the CEC is due largely to SOM (Mendes et al. 2014). It is therefore not surprising that samples with high amounts of clay and/or organic matter typically have higher CEC than sandy soils. ECEC showed significant correlations with Mn_d_, Mn_o_, %clay, %silt and %sand. Extractable Fe and Mn oxides also showed significantly high and positive correlations with %clay and %silt and negative correlations with %sand. Some of these observations have been made by Agboola and Corey (1973), by Inoue et al. (2006) for tropical soils and by Weber et al. (2004) for temperate soils.

**Table 5:** Pearson correlation coefficients (*r*) and test of significance (*n* = 18)

	pH	SOC	Ca	ECEC	Fe_d_	Al_d_	Mn_d_	Fe_o_	Al_o_	Mn_o_	ill	kao	Clay	Silt	Sand	K_f_ DIU	*K*_f_ LIN	*K*_f_ MON	*K*_f_ CLT	*K*_f_ IPU
pH	1.00																			
SOC	0.55^a^	1.00^c^																		
Ca	0.71^c^	0.74^c^	1.00																	
ECEC	0.64^b^	0.82^c^	0.92^c^	1.00																
Fe_d_	0.41	0.51^a^	0.36	0.41	1.00															
Al_d_	0.50^a^	0.38	0.20	0.23	0.55^a^	1.00														
Mn_d_	0.42	0.70^c^	0.49^a^	0.53^a^	0.94^c^	0.43	1.00													
Fe_o_	0.33	0.70^c^	0.45	0.40	0.57^a^	0.20	0.74^a^	1.00												
Al_o_	0.04	0.05	− 0.13	− 0.15	0.57^a^	0.39	0.51	0.29	1.00											
Mn_o_	0.56^a^	0.88^c^	0.66^b^	0.62^b^	0.47	0.42	0.68^a^	0.82^c^	0.19	1.00										
ill	0.25	0.02	0.33	0.20	− 0.22	− 0.08	− 0.21	− 0.23	− 0.08	− 0.07	1.00									
kao	− 0.19	0.01	− 0.30	− 0.17	0.30	0.21	0.27	0.18	0.18	0.10	− 0.98^c^	1.00								
%clay	0.62^b^	0.81^c^	0.59^b^	0.70^c^	0.71^c^	0.60^a^	0.75^c^	0.56^a^	0.04	0.69^c^	− 0.22	0.29	1.00							
%silt	0.77^c^	0.82^c^	0.75^c^	0.79^c^	0.67^c^	0.49	0.70^c^	0.55^a^	− 0.01	0.64^c^	0.08	− 0.04	0.89^c^	1.00						
%sand	− 0.75^c^	− 0.82^c^	− 0.75^c^	− 0.79^c^	− 0.63^c^	− 0.46	− 0.67^c^	0.52^a^	0.05	− 0.62^c^	− 0.09	0.05	− 0.88^c^	− 0.99^c^	1.00					
*K*_f_ DIU	0.63^b^	0.85^c^	0.80^c^	0.82^c^	0.31	0.27	0.46	0.60^b^	− 0.15	0.80^c^	− 0.04	0.03	0.69^a^	0.73^c^	− 0.75^b^	1.00				
*K*_f_ LIN	0.63^b^	0.90^c^	0.80^c^	0.82^c^	0.36	0.30	0.54^a^	0.65^c^	− 0.10	0.85^c^	− 0.06	0.06	0.73^b^	0.75^c^	− 0.76^b^	0.99	1.00			
*K*_f_ MON	0.55^a^	0.79^c^	0.74^c^	0.72^c^	0.15	0.25	0.35^a^	0.59^b^	− 0.16	0.84^a^	− 0.01	0.00	0.58^a^	0.57^a^	− 0.57	0.94	0.94	1.00		
*K*_f_ CLT	0.58^a^	0.92^c^	0.78	0.82^c^	0.41	0.31	0.60^a^	0.68^c^	− 0.01	0.87^c^	− 0.09	0.11	0.73^c^	0.74^c^	− 0.75^c^	0.97	0.99	0.91	1.00	
*K*_f_ IPU	0.51^a^	0.83^c^	0.81^c^	0.80^c^	0.31	0.15	0.50^a^	0.59^b^	− 0.24	0.76^c^	− 0.05	0.03	0.64^c^	0.71^c^	− 0.73^b^	0.92	0.94	0.87	0.94	1.00
*K*_d_ DIU	0.70^c^	0.89^c^	0.82^c^	0.84^c^	0.34	0.31	0.49^a^	0.60^b^	− 0.13	0.82^c^	0.08	− 0.07	0.71^c^	0.79^c^	− 0.80^b^	0.98^c^	0.98	0.93	0.96	0.92
*K*_d_ LIN	0.64^b^	0.91^c^	0.80^c^	0.82^c^	0.37	0.33	0.56^a^	0.66^c^	− 0.07	0.88^c^	− 0.03	0.04	0.74^c^	0.76^c^	− 0.77^b^	0.97	0.99^c^	0.94	0.99	0.94
*K*_d_ MON	0.57^a^	0.88^c^	0.78^c^	0.79^c^	0.26	0.29	0.47^a^	0.64^b^	− 0.13	0.88^c^	− 0.02	0.02	0.67^c^	0.67^c^	− 0.68^b^	0.97	0.98	0.98^c^	0.97	0.93
*K*_d_ CLT	0.59^b^	0.94^c^	0.79^c^	0.83^c^	0.42	0.32	0.61^a^	0.67^c^	− 0.02	0.88^c^	− 0.05	0.07	0.74^c^	0.76^c^	− 0.77^b^	0.95	0.98	0.91	1.00^c^	0.94
*K*_d_ IPU	0.51^a^	0.90^c^	0.79^c^	0.82^c^	0.34	0.21	0.54^a^	0.61^b^	− 0.18	0.81^c^	− 0.03	0.03	0.69^c^	0.74^c^	− 0.76^b^	0.94	0.96	0.89	0.96	0.98^c^

Lowercase letter a, b and c indicate significance at *p* < 0.05, *p* < 0.01 and *p* < 0.001, respectively

**Table 6:** Pearson correlation coefficients (*r*) of *K*_f_ and *K*_d_ with PUH molecular descriptors

	log *K*_ow_	Mw	*α*	*S*	log *S*	log *α*
log *K*_ow_	1.0					
Mw	0.9^a^	1.0				
*α*	0.8	0.5	1.0			
S	− 0.9^a^	− 0.6	− 0.9	1.0		
log *S*	− 0.9^a^	− 0.6	− 0.9	1.0	1.0	
log *α*	0.8	0.5	1.0	− 0.9	− 0.9	1.0
*K*_f mean_	0.8	1.0^a^	0.2	− 0.4	− 0.4	0.2
*K*_d mean_	0.8	1.0^b^	0.2	− 0.4	− 0.4	0.2
*K*_f min_	0.7	0.9^a^	0.2	− 0.4	− 0.4	0.2
*K*_f max_	0.8	0.9^a^	0.2	− 0.4	− 0.4	0.2
*K*_d min_	0.8	1.0^b^	0.2	− 0.4	− 0.5	0.3
*K*_d max_	0.7	0.9^a^	0.2	− 0.4	− 0.5	0.2

Lowercase letters a, b and c indicate significance at *p* < 0.05, *p* < 0.01 and *p* < 0.001, respectively

*min* minimum, *max* maximum

#### Correlations between sorption coefficients and basic soil parameters

Table 5 also presents the correlations between soil basic properties and sorption equilibria (*K*_f_ and *K*_d_) for the five test compounds. The results showed *K*_f_ and *K*_d_ of the five PUHs having positive significant correlations with pH (*r* = 0.51–70, *p* ≤ 0.034) and Fe_o_ (*r* = 0.60–0.68, *p* < 0.01) and very strong and highly significant correlations with %C_org_ (*r* = 0.83–0.94, *p* = 0.0000), ECEC (*r* = 0.74–0.84, *p* = 0.0000), exchangeable Ca (*r* = 0.74–0.82, *p* < 0.001), Mn_o_ (*r* = 0.76–0.88, *p* = 0.0000), %clay (*r* = 0.69–0.74, *p* < 0.001) and %silt (*r* = 0.71–0.79, *p* = 0.0000). Various studies have underscored the dominance of SOC in the sorption of various organic compounds, especially the non-ionisable ones like PUHs (Coquet and Barriuso 2002; Cooke et al. 2004; Ertli et al. 2004; Inoue et al. 2006; El-Khattabi et al. 2007; Fernandez-Bayo et al. 2008; Tian et al. 2010; El-Nahhal et al. 2013). By the same token, very strong relationships have also been reported between sorption coefficients of PUHs and some other soil parameters such as clay content and pH, in addition to SOC (El-Nahhal et al. 2013). For instance, pH was reported as the second most important factor after SOM, for influencing the adsorption of phenylureas by temperate soils (Coquet and Barriuso 2002). But contrary to this finding, our study has shown that ECEC and amorphous Fe and Mn oxides (i.e. Fe_o_ and Mn_o_) exert higher influence than pH on PUH adsorption by tropical soils. Coquet (2003) and Weber et al. (2004) also reported that sorption of PUHs was correlated with the clay content and the composition of the clay particle for temperate soils. Our study has also confirmed this and has generally underscored the importance of soil minerals in sorption of non-ionisable herbicides by low organic matter soils such as the ones used in this study.

#### Correlation with PUH properties

Out of the four PUH molecular properties checked for correlation, only log *K*_ow_ and Mw showed very strong correlations with all the variants of *K*_f_ and *K*_d_ of the compounds studied (Table 6). Mw, in particular, showed a significant correlation (*p* ≤ 0.01). Normally with the trends of both log *K*_ow_ and molecular mass (Mw), the order of the sorption coefficients expected was LIN > DIU > CLT > IPU > MON. Surprisingly however, results in this study showed a slight deviation, with MON which has the least log *K*_ow_ and Mw having higher *K*_f_ and *K*_d_ values than IPU. Therefore, the order of the sorption coefficients (*K*_f_ and *K*_d_) followed the sequence LIN > DIU > CLT > MON > IPU. Except for this inversion between MON and IPU, our results could have followed strictly the trend observed by Fouque-Brouard and Fournier (1996), Weber et al. (2004) and Blondel et al. (2013) for temperate soils. Other PUH molecular descriptors considered (i.e. polarisability index and solubility, and their log transformed values) did not show any strong relationships with the sorption equilibria. This is in agreement with the findings of Theng (1974) for solubility and Hance (1965) for polarisability.

Generally, the increase in adsorption with an increase in the molecular weight has been explained by Traube (Kipling 1965). According to him, as the molecular weight of the adsorbate increases, the surface activity also increases, leading to displacement of more water molecules, with the larger molecules having more points of contact with the adsorbent. The overall effect of this is net entropy and net enthalpy gains. Exception to this rule is found in the case of high molecular weight compounds such as polymers. In the same vein, the increase in adsorption with an increase in log *K*_ow_ is adduced to hydrophobic interactions between the PUH and the organic moiety of the soil. Even though much of the soil organic moiety (in soil humus) is not electrically charged, nevertheless, the PUHs which are themselves uncharged (non-ionic) can react strongly with the uncharged moiety through van der Waals interactions. Although the van der Waals interaction between two molecules is always very weak, when many molecules in a polymeric structure like humus interact simultaneously, the van der Waals interaction creates a synergy and therefore becomes strong, often much stronger than the interaction between PUHs and soil water. This results in their adsorption from the soil solution by humus. According to Sposito (2008), this occurs for two distinct reasons: hydrophobic effect and the presence of non-polar moieties in SOM. However, hydrophobic effect can be exacerbated by the presence of electron withdrawing substituents, especially chlorine. Chlorine is a highly electronegative atom that, upon replacing H atom on a carbon atom, withdraws significant electron charge density carbon-carbon bonds in chain or ring structures, thus rending them less polar and more hydrophobic.

Consequently, LIN and DIU which have two chlorine atoms each (Table 1) recorded higher *K*_f_ and *K*_d_ values than MON and CLT which have one, while MON and CLT recorded higher sorption than IPU that has none. These observations have also been made by Blondel et al. (2013). On the other hand, the difference in the adsorption coefficients of LIN and DIU seems to be caused by the substituents in R1 (Table 1). The presence of a methoxy group in LIN as against a methyl group in DIU confers higher adsorption on LIN. The same observation was made by Blondel et al. (2013), Fouque-Brouard and Fournier (1996) and Grover (1975). CLT has higher *K*_f_ and *K*_d_ values than MON, despite both having one chlorine each on the phenyl ring. This is a result of (i) the difference in the position of the chlorine atom (i.e. *meta* in MON and *para* in CLT). A substituent in *para* position confers higher hydrophobicity (and hence higher adsorption) on a compound than a similar substituent in *meta* position, and (ii) the presence of methyl group in CLT. According to Hance (1965), an increase in *n*-aliphatic chain length or additional aryl substitutions in urea herbicides leads to greater adsorption by soil. In the same token, CLT has higher *K*_f_ value than IPU despite both having the same *K*_ow_. This observation can be attributed to the presence of chlorine atom in the former and the absence of it in the latter, as earlier explained. The observation of Hance (1965) earlier stated is subservient here.

### Comparison of the sorption coefficients in Nigerian soils with published data

To see if our data differ remarkably from those published in the literature (on both tropical and temperate soils), we presented (where available) the reported ranges and means of *K*_f_, *K*_d_ and *K*_oc_ for the five compounds in comparison with the results of this study (Table 7). This discussion will however be limited to comparison of the SOC normalised sorption coefficient (*K*_oc_) values since they are even derived from *K*_d_ (most often) or *K*_f_. For DIU, our values (376–870) fall outside the range 543–956 reported by PPDB (2018) for European soils and the range 107.6–247.5 reported for Brazilian soils by Inoue et al. (2006). They however fall within the ranges 55.3–962 and 145–2.631 reported for tropical soils of Sri Lanka and Brazil, respectively, by Liyange et al. (2006) and Mendes et al. (2014). Our mean *K*_oc_ value of 609 is strikingly different from all reported means, for either temperate or tropical soils, but falls within the range of the reported means (i.e. 480–813 for temperate soils and 407–989 for tropical soils). For LIN, our values (489–1078) fall within the range 547–1159 reported by PPDB (2018). However, the reported means are also different, i.e. 782 (this study) against 843 (PPDB 2018). PPDB (2018) reported a mean *K*_oc_ value of 150 for MON which is considerably lower than our value (237). Our values (215–527; mean, 369) for CLT are quite higher compared to 110–368 and 108–384 (mean, 196) reported by both Elgouzi et al. (2012) and PPDB (2018), respectively, for temperate soils. The same observation could be made about IPU: we reported the value 85–302 (mean, 162), while Elgouzi et al. (2012) reported 54–244. This comparison is however not comprehensive.

**Table 7:** Comparison of present study data with some literature data for both temperate and tropical soils

PUH	*K*_f_			*K*_d_			*K*_oc_		
	Temperate	Tropical	Present study	Temperate	Tropical	Present study	Temperate	Tropical	Present study
DIU	5.2–11.1 (na)^a^		5.3–18.9 (9.0/18)	na (7.37/120)^b^	0.5–75 (9.6/43)^c^	3.5–15.5 (6.6/18)	na (813/na)^d^	55–962 (407/43)^c^	375.6–870 (609/18)
	na (22.2)^e^			na (8.30/na)^d^	1.4–17.1 (8.85/16)^d^		543–956 (680/5)^f^	1453 (989/16)^d^	
	4.5–12.5 (7.0/5)^f^	1.5–2.1 (na/6)^g^		7.7–20.4 (12.8/5)^f^	1.2–2.4 (na/6)^g^			108–248 (na/6)^g^	
LIN	9–12 (na/2)^a^		5.9–28.1 (11.2/18)	na (9.5/43)^b^		4.6–25.5 (8.7/18)	547–1159 (843/4)^f^		489.4–1078.3 (782.1/18)
	na (63.7)^e^	–		11.5–21.9 (15.7/4)^f^	–			–	
	6.3–19.4 (10.4/4)^f^								
	3.0–12.0 (na/3)^f^								
MON	1.7–4.2 (na/2)^a^	–	1.7–9.4 (3.7/18)	na (2/26)^b^	–	1.2–7.0 (2.6/18)	na (150/na)^f^	–	139.8–367.8 (237.5/18)
	na (6.9/nap)^e^								
CLT	3.7–7.3 (na/2)^a^		2.8–15.8 (5.7/18)	1.3–13.9 (na/5)^h^		2.0–13.1 (4.2/18)	110–368 (na/5)^h^		214.5–527.3 (369.1/18)
	1.4–18.6 (na/5)^h^	–		1.2–27.2 (5.9/6)^f^	–		108–384 (196/6)^f^	–	
IPU	4.0–6.4 (na/2)^a^		1.1–5.9 (2.4/18)	0.6–7.7 (na/5)^h^	–	0.9–4.6 (1.7/18)	54–244 (na/5)^h^	–	84.6–301.7 (162/18)
	0.6–10.1 (na/5)^h^								
	0.3–27.1 (2.8/2)^f^	–							
	na (3.0/33)^f^								

Report format: range of values or single value (reported mean/number of soils used for the study)^reference^

*na* not available (where values were not reported), *nap* not applicable (where reported values were single values)

^a^Blondel et al. (2013)

^b^Weber et al. (2004)

^c^Liyange et al. (2006)

^d^Mendes et al. (2014)

^e^Wang et al. (2015)

^f^PPDB (2018)

^g^Inoue et al. (2006)

^h^Elgouzi et al. (2012)

## Conclusion

It is indisputable that an understanding of the sorption mechanism is fundamental for predicting the fate of organic contaminants in the environment. With respect to pesticide-soil interactions, the environmentally essential distribution of phenylurea herbicides between soil and soil water has always been estimated by measuring *K*_f_ or *K*_d_ values. While there exists relatively large data of sorption coefficients of some of the PUHs in temperate soils, there are just very few data on sorption equilibria of these compounds in tropical soils. More so, those few by Liyange et al. (2006), Inoue et al. (2006) and Mendes et al. (2014) were only on diuron. It is therefore very difficult to make valid comparison with other data from tropical soils. Nevertheless, the attained ranges of sorption data for DIU and LIN in this study were within the ranges reported for temperate soils, while those of MON, CLT and IPU were outside the ranges obtained from temperate soils. Thus, our hypothesis that PUHs sorbed differently to tropical and temperate soils can only be said to be partly confirmed. Oliver et al. (2005) which compared sorption of diuron in Australian (temperate) soils and Filipino (tropical) soils also concluded that they were the same. Therefore, to answer the question whether the existing data on sorption coefficients of this group of herbicides from temperate soils can be extrapolated for tropical soils, more studies and/or a larger database on their sorption coefficients in tropical soils may be needed. This was also the conclusion of Liyange et al. (2006). From correlation analysis, this study has shown that not only SOM but also CEC and soil mineral fractions (i.e. clay/silt and amorphous Fe and Mn oxides) are important soil parameters that largely explain the variations in PUH sorption by tropical soils, with SOM being the most prominent. Particularly, our study has shown hitherto unrevealed correlation (in either temperate or tropical soils) between PUH adsorption and amorphous Mn oxides. Lastly, as expected, the sorption coefficients showed high correlations (0.8) with log *K*_ow_, but much higher and significant correlations (1.0) with Mw. Thus, the molecular mass of these compounds would be a better predictor for their sorption behaviour than log *K*_ow_.

## Electronic supplementary material

ESM 1 (DOCX 91 kb)
