# Supplementary material for: Adsorption of phenylurea herbicides by tropical soils
Source: Environ Monit Assess. 2020 Mar 4;192(4):212. doi: 10.1007/s10661-020-8160-2 (PMC7056688; doi:10.1007/s10661-020-8160-2)
Supplement: Supplementary file 1 — (DOCX 91 kb) [file 10661_2020_8160_MOESM1_ESM.docx]

SM1

|  |
| --- |

Pseudo-second order kinetic parameters

|  | linuron | | | | | diuron | | | | | monuron | | | | |
| --- | --- | --- | --- | --- | --- | --- | --- | --- | --- | --- | --- | --- | --- | --- | --- |
|  | R^2^ | Q_e model_ | Q_e expt_ | h | k_2_ | R^2^ | Q_e model_ | Q_e expt_ | h | k_2_ | R^2^ | Q_e model_ | Q_e expt_ | h | k_2_ |
| Akn | 0,998 | 83,3 | 86,7 | 95,1 | 13,7 | 0,985 | 60,2 | 86,1 | 11,7 | 3,2 | 1,000 | 15,6 | 40,1 | 5,0 | 20,5 |
| Apm | 0,997 | 65,8 | 62,1 | 4,9 | 1,1 | 0,997 | 53,5 | 64,0 | 0,7 | 0,3 | 0,993 | 14,0 | 39,7 | 0,9 | 4,8 |
| Asj | 0,999 | 81,3 | 78,4 | 98,0 | 14,8 | 0,995 | 50,3 | 70,0 | 15,7 | 6,2 | 0,994 | 19,3 | 42,5 | 0,8 | 2,2 |
| Bkt | 1,000 | 96,2 | 79,7 | 14,4 | 1,6 | 1,000 | 63,7 | 72,6 | 9,0 | 2,2 | 0,988 | 21,0 | 50,4 | 9,0 | 20,4 |
| Idt | 0,999 | 67,6 | 74,4 | 7,6 | 1,7 | 0,984 | 59,9 | 55,8 | 1,1 | 0,3 | 0,984 | 15,8 | 26,7 | 0,9 | 3,5 |
| Mtk | 0,998 | 76,3 | 77,3 | 9,8 | 1,7 | 0,998 | 43,1 | 65,0 | 5,3 | 2,8 | 0,991 | 11,8 | 38,7 | 2,0 | 14,2 |
| Skn | 0,999 | 97,1 | 97,1 | 10,2 | 1,1 | 1,000 | 59,5 | 84,4 | 12,3 | 3,5 | 0,999 | 20,1 | 50,6 | 2,8 | 6,8 |
| Uib | 1,000 | 126,6 | 132,3 | 23,3 | 1,5 | 0,999 | 89,3 | 121,0 | 71,4 | 9,0 | 0,999 | 35,7 | 79,6 | 5,3 | 4,2 |
| Ibd | 0,995 | 62,9 | 60,8 | 50,0 | 12,6 | 0,999 | 43,7 | 41,7 | 2,0 | 1,0 | 0,958 | 12,2 | 23,6 | 0,2 | 1,4 |
| Gbn | 1,000 | 79,4 | 87,2 | 27,5 | 4,4 | 0,990 | 62,5 | 73,4 | 1,0 | 0,3 | 0,999 | 18,3 | 41,1 | 1,3 | 3,8 |
| Iwo | 1,000 | 72,5 | 75,1 | 30,6 | 5,8 | 0,981 | 50,0 | 69,9 | 1,7 | 0,7 | 0,989 | 16,7 | 29,1 | 0,9 | 3,4 |
| Odd | 0,999 | 67,6 | 57,9 | 9,6 | 2,1 | 0,960 | 49,5 | 41,9 | 0,4 | 0,2 | 0,995 | 8,7 | 23,6 | 0,4 | 5,2 |

k_2_: kinetic rate constant (Kgmg^-1^ min^-1^), h: initial adsorption rate (mgKg^-1^ min^-1^), Q_e_ expt (Q_e_ experimental)

SM2

a)

b)

c)

d)

e)

f)

g)

h)

i)

j)

Isotherm plots: (a) linuron for SOC ˂ 1%, (b) linuron for SOC ≥ 1%, (c) diuron for SOC ˂ 1%, (d) diuron for SOC ≥ 1%, (e) monuron for SOC ˂ 1%, (f) monuron for SOC ≥ 1%, (g) chlorotoluron for SOC ˂ 1%, (h) chlorotoluron for SOC ≥ 1%, (i) isoproturon for SOC ˂ 1%,( j) isoproturon for SOC ≥ 1%

SM3

Linear plots of K_d.avrg_ vs f_oc_ for the five compounds
